# Annual Wellness Visits, Cognitive Function, and Early Dementia Diagnosis ‐ Analysis of the Health Retirement Study linked to Medicare Data

**DOI:** 10.1002/alz70860_097062

**Published:** 2025-12-23

**Authors:** Yong‐Fang Kuo, Efstathia Polychronopoulou, Ming Tzeng, Sadaf Arefi Milani, Mukaila A Raji

**Affiliations:** ^1^ University of Texas Medical Branch, Galveston, TX, USA

## Abstract

**Background:**

Prior work using administrative claims data has found an association of annual wellness visits (AWVs) with early diagnosis of mild cognitive impairment (MCI) among Medicare beneficiaries. Adding cognitive function assessment from surveys before and after AWV will help better understand this association.

**Method:**

This population‐based cohort study used Health Retirement Survey (HRS) linked to Medicare from 2011 to 2019. For each study year (2013, 2015, 2017, and 2019), we selected subjects with an initial AWV in the year, no prior MCI or Alzheimer's Disease Related Dementia (ADRD) diagnosis (from Medicare), and cognitive assessments (from HRS) both in the year before and after AWV (*n* = 1585). Through propensity score matching, the subjects without AWVs at each study year were selected (*n* = 2757). All covariates were well balanced including pre‐visit cognitive assessment. The primary outcomes were the post‐visit cognitive assessment, measured by the Langa‐Weir Classification [normal cognition, cognitive impairment no dementia (CIND) and probable dementia], and first MCI or ADRD diagnosis by ICD9 and ICD10 codes from Medicare inpatient and outpatient claims within 2 years after AWV.

**Result:**

Among subjects (*n* = 235) diagnosed with MCI/ADRD within 2 years, the AWV group had proportionally more MCI diagnoses (26% vs 20%) than the non‐AWV. AWV visit was significantly associated with increase in MCI diagnosis (HR: 1.79, 95% CI: 1.10 – 2.91) and insignificantly associated with increase in ADRD diagnosis (HR: 1.23, 95% CI: 0.91‐ 1.66). Subjects classified as CIND and probable dementia from cognitive assessment prior to AWV, were strongly associated with ADRD diagnosis within 2 years (10% and 19%) compared to those with ‘normal’ assessment (2.8%). The interaction between AWV and prior cognitive assessment was not significantly associated with MCI or ADRD. However, among those diagnosed with ADRD, lower proportion of patients were classified as “probable dementia” (from cognitive assessment) before diagnosis in AWV group (8.8%) than non‐AWV group (15.1%); and higher proportion of patients were “probable dementia” after diagnosis in AWV group (40.5%) than non‐AWV group (31.6%).

**Conclusion:**

This study suggests that receipt of a Medicare AWV is associated with increased identification of early cognitive impairment from both clinician‐based diagnosis and survey‐based cognitive assessment.

